# Mechanisms underlying 3-bromopyruvate-induced cell death in colon cancer

**DOI:** 10.1007/s10863-015-9612-1

**Published:** 2015-06-09

**Authors:** Yiming Sun, Zhe Liu, Xue Zou, Yadong Lan, Xiaojin Sun, Xiu Wang, Surong Zhao, Chenchen Jiang, Hao Liu

**Affiliations:** Faculty of Pharmacy, Bengbu Medical College, Bengbu, 233000 Anhui People’s Republic of China; Department of Surgical Oncology, The First Affiliated Hospital of Bengbu Medical College, Bengbu, 233004 People’s Republic of China; School of Medicine and Public Health, Faculty of Health, University of Newcastle, Newcastle, NSW Australia

**Keywords:** 3-Bromopyruvate, ATP, Apoptosis, Autophagy, Necroptosis

## Abstract

3-Bromopyruvate (3BP) is an energy-depleting drug that inhibits Hexokinase II activity by alkylation during glycolysis, thereby suppressing the production of ATP and inducing cell death. As such, 3BP can potentially serve as an anti-tumorigenic agent. Our previous research showed that 3BP can induce apoptosis via AKT /protein Kinase B signaling in breast cancer cells. Here we found that 3BP can also induce colon cancer cell death by necroptosis and apoptosis at the same time and concentration in the SW480 and HT29 cell lines; in the latter, autophagy was also found to be a mechanism of cell death. In HT29 cells, combined treatment with 3BP and the autophagy inhibitor 3-methyladenine (3-MA) exacerbated cell death, while viability in 3BP-treated cells was enhanced by concomitant treatment with the caspase inhibitor benzyloxycarbonyl-Val-Ala-Asp fluoromethylketone (z-VAD-fmk) and the necroptosis inhibitor necrostatin (Nec)-1. Moreover, 3BP inhibited tumor growth in a SW480 xenograft mouse model. These results indicate that 3BP can suppress tumor growth and induce cell death by multiple mechanisms at the same time and concentration in different types of colon cancer cell by depleting cellular energy stores.

## Introduction

Colorectal cancer (CRC) is the third most diagnosed cancer worldwide, and there has been extensive research on anti-tumorigenic drugs for its treatment (Jemal et al. 2011). CRC is typically treated by surgical resection and postoperative adjuvant radio- and chemotherapy. However, the latter is associated with strong side effects and dose-limiting toxicity. As such, there is ongoing need for developing less toxic but still effective drugs to treat CRC.

The Warburg effect posits that tumor cells depend on glycolysis to generate ATP even in the presence of oxygen (Warburg 1956). The enzyme hexokinase (HK), a key glycolytic enzyme, is overexpressed in tumor cells and is inhibited by 3-bromopyruvate (Cardaci et al. 2012; da-Silva et al. 2004). It is putative that proton-linked monocarboxylate transporters (MCTs) mediate 3BP uptake. 3BP mediate pyruvylation of GAPDH which decrease ATP and anabolic precursors. Pyruvylated GAPDH could elicite the pro-apoptotic effects after translocating into the nucleus phenocoping (Cardaci et al. 2012). Inhibition of hexokinase-II by 3BP decreases ATP and NADPH levels, thereby blocking glycolysis in tumor cells and slowing or halting their growth (Chen et al. 2009; Chesney et al. 1999; Galluzzi et al. 2012). 3-BrPA mediated impairment of succinate dehydrogenase (SDH) activity lowers succinate-derived ATP levels and increases ROS production (Cardaci et al. 2012).

Previous study (Leist et al. 1997) indicates that apoptosis is a process depending on ATP, the lack of ATP would result in cell necrosis. Research (Xu et al. 2005) reported cell deaths by 3BP contain apoptosis and necrosis. While massive depletion of ATP leads to necrosis, a moderate decrease leads to induction of cellular autophagy to overcome situations of energetic or metabolic stress (Shoshan 2012). The absence of ATP leads to DNA degradation and ultimately to cell death (Parks et al. 2013). Moreover, mitochondrial permeability is increased by inhibiting HK and the consequent release of cytochrome C activates caspases that induce cellular apoptosis (Ferraro et al. 2008; Zuo et al. 2011). Under apoptosis-deficient conditions or upon infection by certain types of virus, receptor-interacting protein (RIP)1 forms a complex with RIP3 to mediate necroptosis, which is characterized by the activation of autophagy with necrotic features. The formation of this complex requires RIP1 kinase activity, which is inhibited by the small molecule Nec-1 (Degterev et al. 2005; Yuan and Kroemer 2010).

Three forms of cell death are recognized, including apoptosis, cell death associated with autophagy, and necrosis. The latter is typically unregulated, whereas apoptosis and autophagic cell death are considered as two types of programmed cell death (Galluzzi et al. 2012). However, an increasing number of studies have demonstrated that necrosis can also be regulated in a process known as necroptosis (Cho et al. 2009; He et al. 2009; Zhang et al. 2009a). Morphological features of apoptotic cells are chromatin condensation leading to pyknotic nuclei (Schweichel and Merker 1973) and the close packing of cytoplasmic organelles resulting from a loss of cytosol (Bellairs 1961). Autophagic cells are characterized by the appearance of autophagic vacuoles in the cytoplasm. The conversion of microtubule-associated protein 1 light chain (LC)3-Ito LC3-IIin the membranes of autophagic bodies is widely used as an autophagosome marker (Kabeya et al. 2000; Tanida et al. 2004; Zhang et al. 2014). Autophagy can be specifically inhibited by 3-MA. Necroptotic cells exhibit a breakdown of the cell membrane and swelling of cellular organelles (He et al. 2009).

Our previous work found that 3BP can induce apoptosis via AKT signaling in breast cancer cells (Liu et al. 2014). This was investigated in the present study using SW480 and HT29 human colon carcinoma cell lines. The results showed that 3BP induces multiple forms of cell death by energy depletion in vitro, which is beneficial to reduce the resistance of the single cell death form by drugs, providing a potential treatment for a special cells, and 3BP possesses anti-tumor ability in vivo, suggesting that 3BP can be used in the treatment of CRC.

## Materials and methods

### Reagents and antibodies

The compounds 3BP, 3-MA, 3-(4,5-dimethylthiazol-2-yl)-2,5-diphenyltetrazolium bromide (MTT), and Nec-1 were purchased from Sigma-Aldrich (St. Louis, MO, USA). The caspase inhibitor z-VAD-fmk was purchased from Calbiochem (San Diego, CA, USA). The JC-1(5,5′,6,6′-tetrachloro-1,1′,3,3′-tetraethyl-benzimidazolylcarbocyanine iodide) and PI assay kits were purchased from Beyotime Institute of Biotechnology (Wuhan, China) and the ATP Assay kit was from Merck KGaA (Darmstadt, Germany) and An Annexin V FITC/propidium iodide (PI) apoptosis detection kit was purchased from Nanjin KeyGen Biotech (Nanjing, China). The enzyme-linked immunosorbent assay kit for Hexokinase II was purchase from Cloud-Clone Crop (Houston, TX, USA). The following antibodies were used: rabbit monoclonal antibodies against X-linked inhibitor of apoptosis protein (XIAP), cellular inhibitor of apoptosis protein (cIAP)1 (both from Bioss, Hong Kong, China), and cIAP2 (Abgent, Suzhou, China); monoclonal antibodies against LC3, Bcl-2-associated X protein (Bax), myeloid cell leukemia (Mcl)-1, and B cell CLL/Lymphoma (Bcl)-2 protein (all from Proteintech, Chicago, IL, USA); and rabbit monoclonal antibodies against RIP1 and 3 and β-actin and HKII(Santa Cruz Biotechnology, Santa Cruz, CA, USA); Anti-monocarboxylate transporter (MCT)1 antibody and rabbit anti-monocarboxylate transporter (MCT)4 were obtained from Millipore (Temecula, CA, USA); Anti-monocarboxylate transporter (MCT)2 was form Abcam (Cambridge, MA, USA). All reagents were dissolved according to the manufacturer’s instructions.

### Cell culture

Human colon carcinoma SW480 and HT29 cells were obtained from the American Type Culture Collection (Manassas, VA, USA) and were passaged for less than half a year. Cells were grown in high-glucose Dulbecco’s Modified Eagle’s Medium (DMEM) supplemented with 10 % fetal bovine serum, 100 U/ml penicillin, and 100 μg/ml streptomycin at 37 °C in 95 % air and 5 % CO_2_.

### MTT assay

Cells were seeded in a 96-well plate at a density of 6 × 10^3^ cells per well and treated with different concentrations of 3BP. At 24, 48, and 72 h, MTT (5 mg/ml in phosphate-buffered saline, PBS) was added for 4 h at 37 °C. After 4 h, the solution was replaced with 150 μl of dimethylsulfoxide (DMSO, Biosharp, Hefei, China) and 30 min later, cell viability was determined by measuring absorbance at a wavelength of 490 nm using a microplate reader (Synergy HT, BioTek, Vermont, U.S.A).

### Colony formation assay

Cells were seeded in 6-well plates at 1 × 10^5^ cells per well and incubated for 24 h, then treated with various concentrations of 3BP for 24 h. The medium was replaced with 2 ml fresh medium and cells were cultured for 5 more days, then fixed with paraformaldehyde for 10 min at −20 °C followed by staining with 2 % crystal violet for 20 min.

### Flow cytometry

Prior to 3BP treatment, 2 × 10^5^ cells per well were seeded in a 12-well plate for 24 h. At exponential growth phase, cells were treated with different concentrations of 3BP (40, 80, 160, and 320 μmol/L) for 24 h, which was followed by PI staining and Accuri C6 flow cytometry (BD Biosciences, State of New Jersey, U.S.A). In addition, annexin-V FITC/PI staining was carried out according to the manufacturer’s instructions to detect cellular apoptosis.

### Nuclear staining

Cells (2 × 10^6^) were seeded in a 6-well plate for 24 h until exponential growth phase, then treated with 3BP (0, 40, 80, 160, and 320 μmol/L) for 48 h. Cells were harvested and washed twice with cold PBS, then fixed with 4 % ethanol for 30 min at room temperature, followed by two washes with PBS and a 1 min incubation in 100 μl of DAPI solution (2 μg/ml). Cell morphology was evaluated using a IX71 fluorescence microscope (Olympus, Tokyo, Japan). Cells showing chromatin condensation and nuclear fragmentation were determined to be apoptotic.

### Determination of ATP

ATP levels were measured using a luminescence-based ATP Assay kit. The assay uses luciferase to catalyze the generation of a fluorescent signal from ATP, luciferin, and oxygen; with the degree of fluorescence proportional to the amount of ATP. Cells (2 × 10^5^) seeded in a 12-well plate for 24 h, were incubated with various concentrations of 3BP for 5 h at 37 °C, with untreated cells serving as a control group. After incubation, cells were collected and homogenized in RIPA lysis buffer for 10 min on ice. Cell lysates were centrifuged at 13,225×*g* for 5 min at 4 °C. 100 μl of nucleotide-releasing buffer per well and 1 μl ATP-monitoring enzyme per well were added to a 96-well plate, 30 μl of each suspension were transferred to each well; and after a 60 s incubation at 25 °C, the signal was measured using a Luminoskan luminometer (Thermo Scientific, Atlanta, GA, USA).

### Determination of mitochondrial membrane potential

Cells were seeded at a density of 2 × 10^5^ cells per well in 12-well-plates and treated with various concentrations of 3BP. After 24 h, changes in mitochondrial membrane potential were determined by staining cells with the cationic dye JC-1 using a kit according to the manufacturer’s instructions. Green and red fluorescence was detected on the 1 and 2 channels, respectively, of an IX71 fluorescence microscope.

### Western blot analysis

Cells were collected and homogenized in RIPA lysis buffer for 30 min on ice. Cell lysates were centrifuged at 13,225×*g* for 30 min at 4 °C. Proteins were separated on a 12 % sodium dodecyl sulfate polyacrylamide gel and transferred to a nitrocellulose membrane (Universal Hood II, BioRad Laboratories, Hercules, CA, U.S.A), which was incubated with primary antibodies overnight at 4 °C followed by the appropriate secondary antibody, with β-actin used as a loading control.

### Enzyme-linked immunosorbent assay kit for Hexokinase II

Cells were seeded at a density of 2 × 10^5^ cells per well in 12-well-plates and treated with various concentrations of 3BP for 24 h at 37 °C, with untreated cells serving as a control group. After incubation, cells were collected and homogenized in 1 % triton-100 for 10 min on ice. Cell lysates were centrifuged at 1000×*g* for 20 min at 4 °C. Detect the concentration of HKII according to the manufacturer’s instructions.

### Evaluation of cell death type by electron microscopy

Cells were fixed with 3 % glutaraldehyde and 2 % paraformaldehyde in 0.1 M PBS (pH 7.4) overnight at 4 °C, then postfixed with 1 % osmium tetroxide for 1.5 h, washed, and stained with 3 % aqueous uranyl acetate for 1 h before dehydration in a graded series of ethanol and acetone and embedding in Araldite. Ultrathin sections were cut on a Reichert ultramicrotome (Leica, Wetzlar, Germany), stained with 0.3 % lead citrate, and analyzed by TEM (Olympus JEOL, Peabody, MA, USA).

### Xenograft model

Female nude mice (BALB/c) 4–5 weeks of age and weighing 18–20 g were purchased from the animal experimental center of Beijing vitalriver and maintained under specific pathogen-free conditions. The experimental protocol was approved by the ethics committee of Bengbu Medical College and was carried out in accordance with the Guidance and Suggestions for the Care and Use of Laboratory Animals published by The Ministry of Science and Technology of China. SW480 cell suspensions with >90 % viability were used for injections. Mice were grafted subcutaneously in the left flank with 10^7^ cells resuspended in 0.2 ml sterile DMEM. Mice were randomized into 3BP (8 mg/kg), DNR positive control (0.8 mg/kg), and PBS negative control groups (*n* = 5 per group) when tumor volume reached approximately 100 mm^3^. Group the implantation model using stratified random grouping according to the tumor size. Tumors were measured with a caliper and volume was calculated using the formula: 0.5 × *a* × *b*^2^, where *a* and *b* are tumor length and width, respectively. Tumors were stored in 4 % formalin solution, embedded in paraffin, and cut into sections that were stained with hematoxylin and eosin (H & E).

### Statistical analysis

Independent experiments were performed in triplicate. Data are expressed as the mean ± SEM of three experiments. SPSS v.16.0 software (SPSS Inc., Chicago, IL, USA) was used for data analysis. Half-maximal inhibitory concentration (IC50) was calculated by probit regression analysis. Mean differences were evaluated by t-test analysis of variance. *p < 0.05 was considered statistically significant.

## Results

### 3BP selectively inhibits colon cancer cell viability

**Fig. 1:**
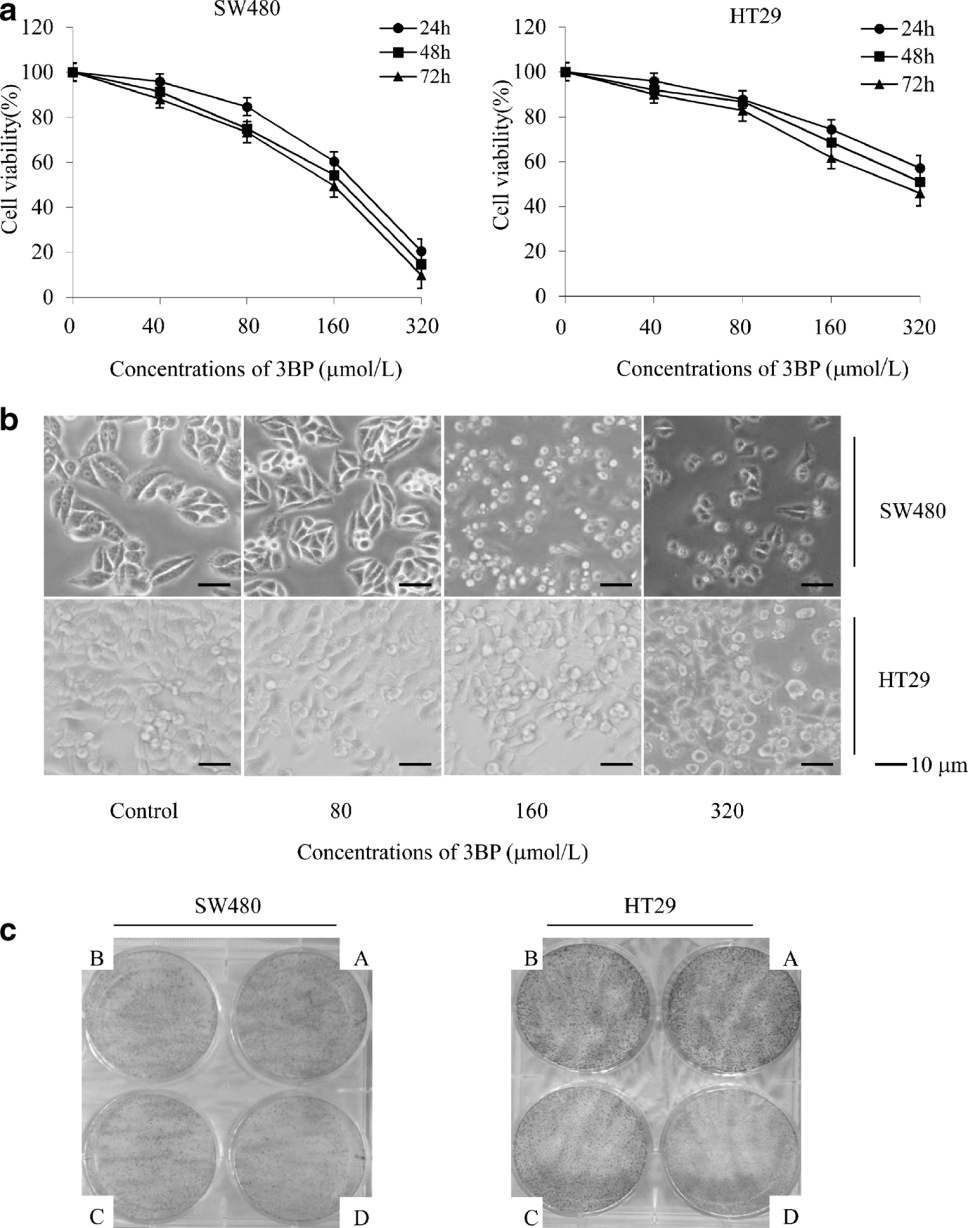
3BP selectively inhibits colon cancer cell viability. **a** SW480 and HT29 cells were treated with 40, 80, 160, or 320 μmol/L 3BP for 24, 48, or 72 h; cell viability was analyzed by the MTT assay. **b** SW480 and HT29 cells were treated with various concentrations 3BP (80, 160, or 320 μmol/L) for 24 h and morphology was examined by light microscopy with differential interference contrast optics. Representative images are shown. **c** SW480 cells were treated with 0 (A), 10 (B), 20 (C), or 30 (D) μmol/L and HT29 cells were treated with 0 (A), 30 (B), 40 (C), or 50 (D) μmol/L 3BP for 5 days. Data represent mean ± SEM of three independent experiments

SW480 cells had greater sensitivity to 3BP than HT29 cells, and viability decreased in a dose-dependent manner (Fig. 1a and b). In addition, colony formation in both cell types was inhibited by increasing concentrations of 3BP (10–50 μmol/L) (Fig. 1c), indicating that 3BP efficiently inhibits colon cancer cell growth.

### 3BP lowers HKII level and mediate MCTs

**Fig. 2:**
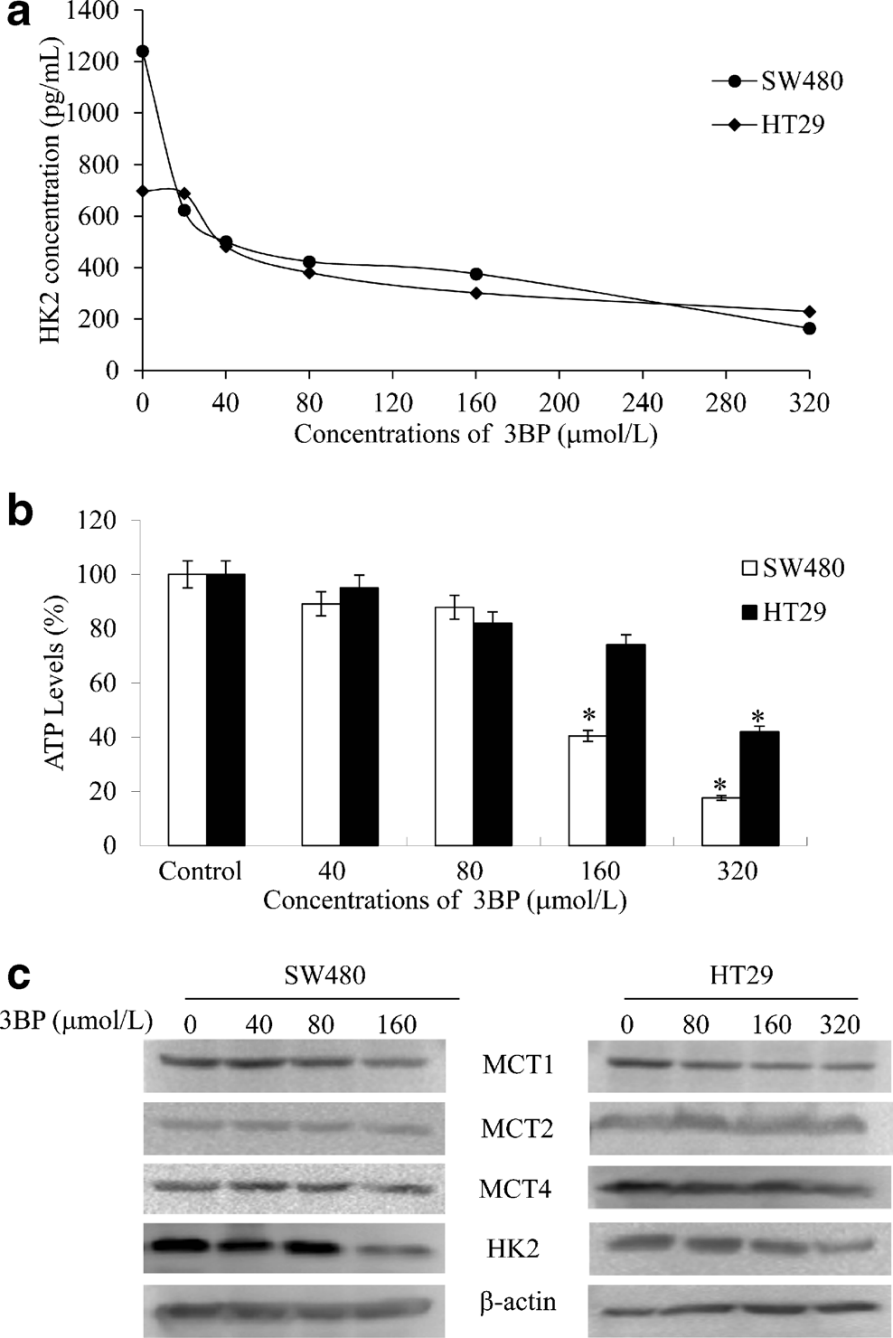
3BP induces HKII and ATP reduction via MCTs transportation in colon cancer cells. **a** SW480 and HT29 cells were treated with indicated concentrations of 3BP for 24 h, and HKII level were assessed by Enzyme-linked immunosorbent assay kit. **b** Intracellular ATP levels were measured in SW480 and HT29 treated with 40, 80, 160, or 320 μmol/L 3BP for 5 h. control. **c** SW480 and HT29 cells treated with various concentrations of 3BP for 24 h were subjected to HKII level and MCTs by Western blot analysis, β-actin served as loading control. Data represent mean ± SEM of three independent experiments. *p < 0.05 vs

ELISA results suggested that HKII was reduced as the 3BP concentration dependence (Fig. 2a). Cellular ATP levels was reduced by treatment with 3BP (Fig. 2b). These data indicate that and induces cell death by decrease ATP level. Western blotting results showed that with the increase of concentrations of 3BP the expression of MCT1, MCT2 and HKIIwas reduced, in turn MCT2 expression did not change basically (Fig. 2c), The results indicated the 3BP transporters into colon cancer cells through MCTs, influencing the expression of HKII.

### 3BP induces apoptosis in colon cancer cells

**Fig. 3:**
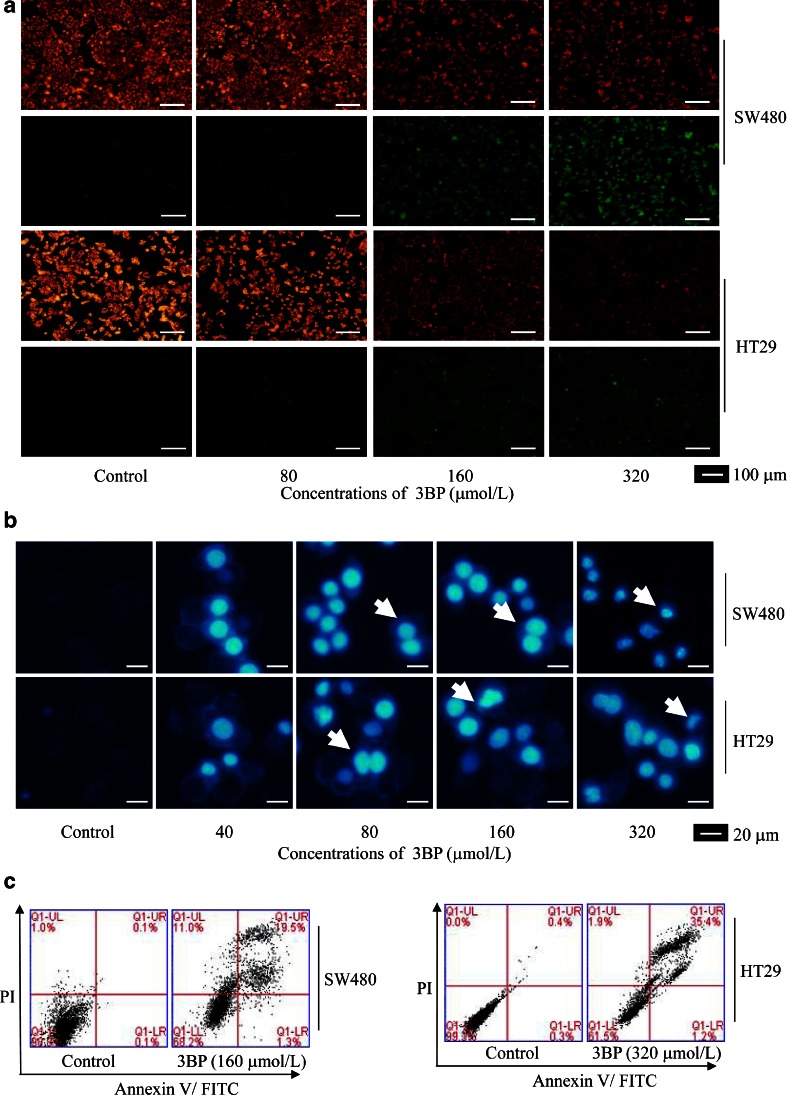
3BP-induced apoptosis in colon cancer cells. **a** SW480 and HT29 cells were treated with indicated concentrations of 3BP for 24 h, and the mitochondrial membrane potential were assessed by JC-1 staining and fluorescence microscopy. **b** SW480 and HT29 cells were treated with indicated concentrations of 3BP for 48 h, and nuclei were stained with DAPI and visualized by fluorescence microscopy. *White arrowheads* indicate apoptotic cells. **c** SW480 and HT29 cells were treated with indicated concentrations of 3BP for 24 h, and cells were determined using flow cytometry of PI/Annexin V-stained cells. Data represent mean ± SEM of three independent experiments

**Fig. 4:**
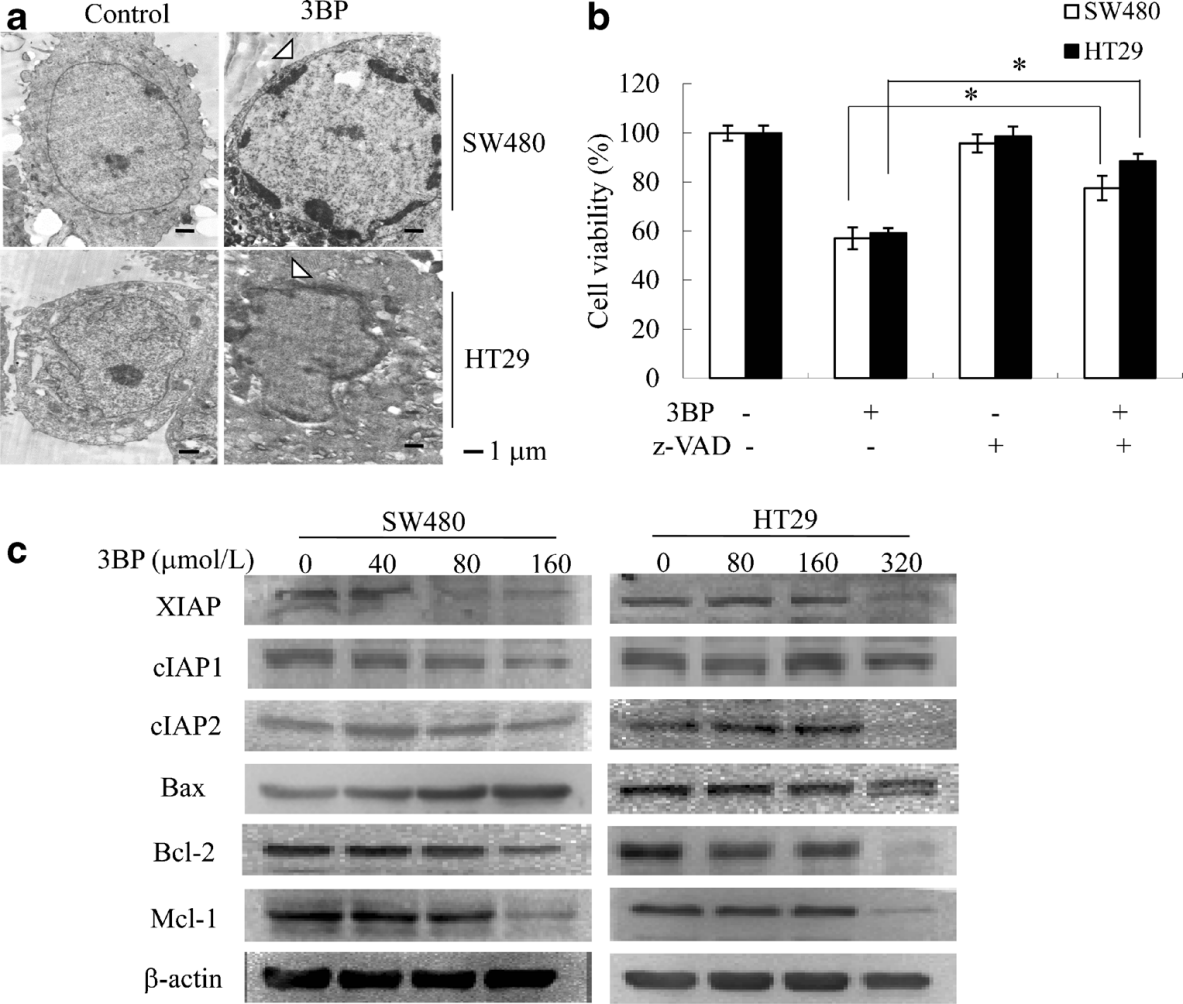
3BP-induced apoptosis in colon cancer cells. **a** Electron microscopy of cells treated for 24 h with Control, 160 or 320 μmol/L 3BP, *white arrowheads* denote chromatin pyknosis in the cells treated with 3BP. **b** Viability of SW480 or HT29 cells treated with DMSO, 3BP (160 or 320 μmol/L), 3BP/z-VAD (20 μmol/L, ) was analyzed by MTT assay. Both cells were pretreated with z-VAD 1 h before treatment with 3BP/z-VAD. **c** SW480 and HT29 cells treated with various concentrations of 3BP for 24 h were subjected to apoptosis-related proteins by Western blot analysis, β-actin served as loading control. Data are representative of three independent experiments,*p < 0.05

JC-1 staining was used to detect mitochondrial membrane potential (Fig. 3a). Treated cells exhibited condensed and fragmented nuclei, which are indicative of apoptosis, and 4,6-diamidine-20-phenylindole dihydrochloride (DAPI) staining (Fig. 3b), Annexin-V FITC/PI staining confirmed that 3BP induced apoptosis in SW480 and HT29 cells (Fig. 3c). To investigate the mechanism by which 3BP induces cell death, TEM examination revealed shrinkage, blebbing, nuclear fragmentation, and chromatin condensation in apoptotic cells (Fig. 4a); these effects were abolished by treatment with the apoptosis inhibitor z-VAD-fmk (Fig. 4b). The induction of apoptosis in colon cancer cells by 3BP was confirmed by the downregulation of XIAP, cIAP1, cIAP2, Mcl-1, and Bcl-2 protein levels and the upregulation of Bax protein level (Fig. 4c).

### 3BP induces necroptosis in colon cancer cells

**Fig. 5:**
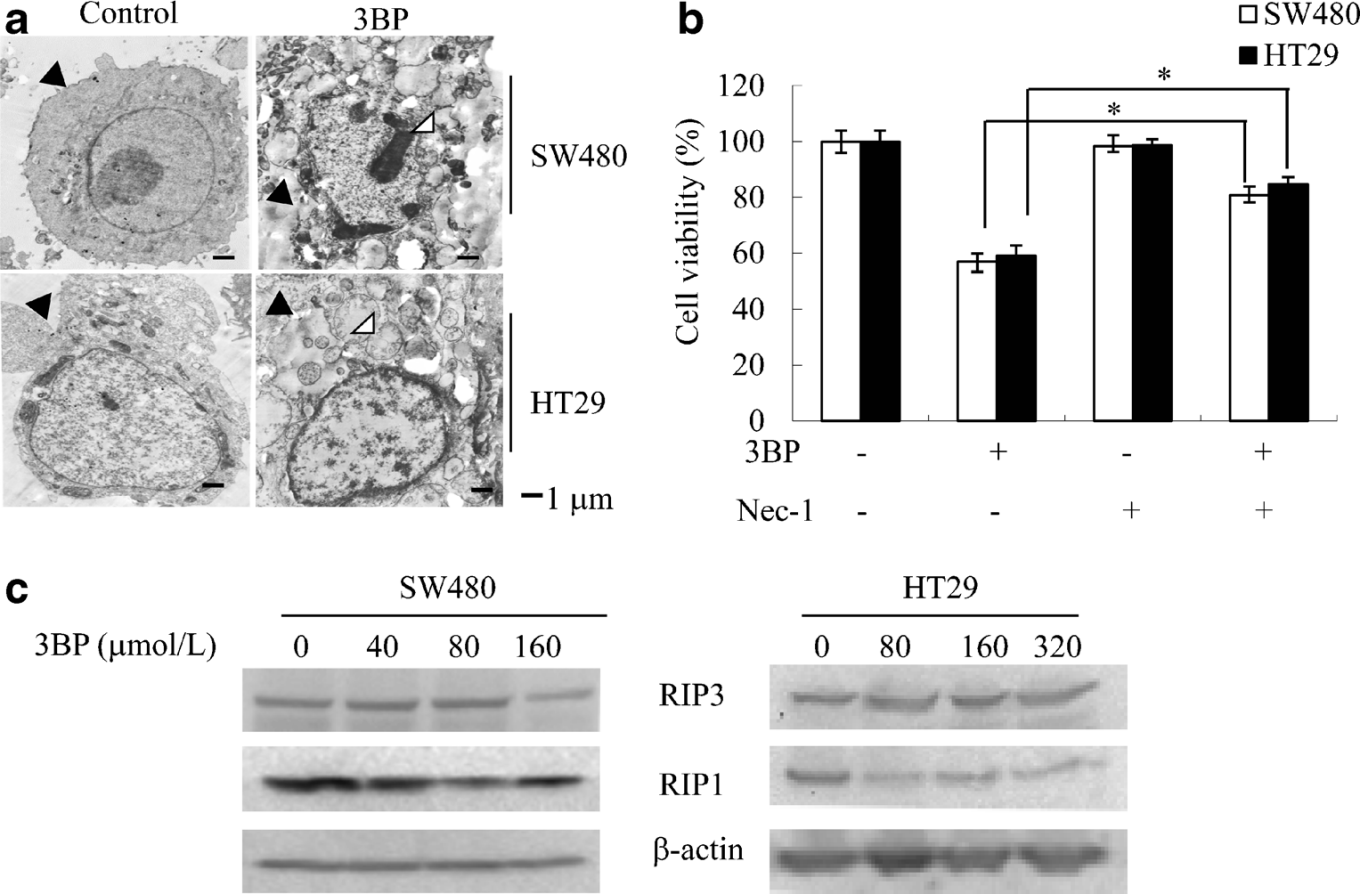
3BP-induced necroptosis in colon cancer cells. **a** Electron micrographs of cells treated for 24 h with 160 or 320 μmol/L 3BP or the vehicle dimethylsulfoxide (DMSO; control). *Black arrowheads* indicate cell membrane integrity in control cells and membrane breakdown in 3BP-treated cells; *white arrowheads* indicate swelling of cellular organelles in 3BP-treated cells. **b** SW480 or HT29 cell viability following treatment with 3BP (160 or 320 μmol/L), 3BP/Nec-1 (20 μmol/L), or DMSO, as analyzed by the MTT assay. Both cells were pretreated with Nec-1 1 h before treatment with 3BP/Nec-1. **c** SW480 and HT29 cells treated with indicated concentrations of 3BP for 24 h were analyzed for the expression of necroptosis-related proteins by western blotting, with β-actin serving as a loading control. Data represent mean ± SEM of three independent experiments, *p < 0.05

Interesting, the TEM analysis revealed the presence of necroptotic cells following 3BP treatment, which are characterized by an absence of typical nuclear fragmentation, organelle (especially mitochondrial) swelling, and loss of plasma membrane integrity (Fig. 5a). The viability of 3BP-treated cells was rescued by application of the necroptosis inhibitor Nec-1 (Fig. 5b), as evidenced by the downregulation of RIP1 protein (Fig. 5c). These data indicate that 3BP induces necroptosis in colon cancer cells.

### 3BP induces autophagy in colon cancer cells

**Fig. 6:**
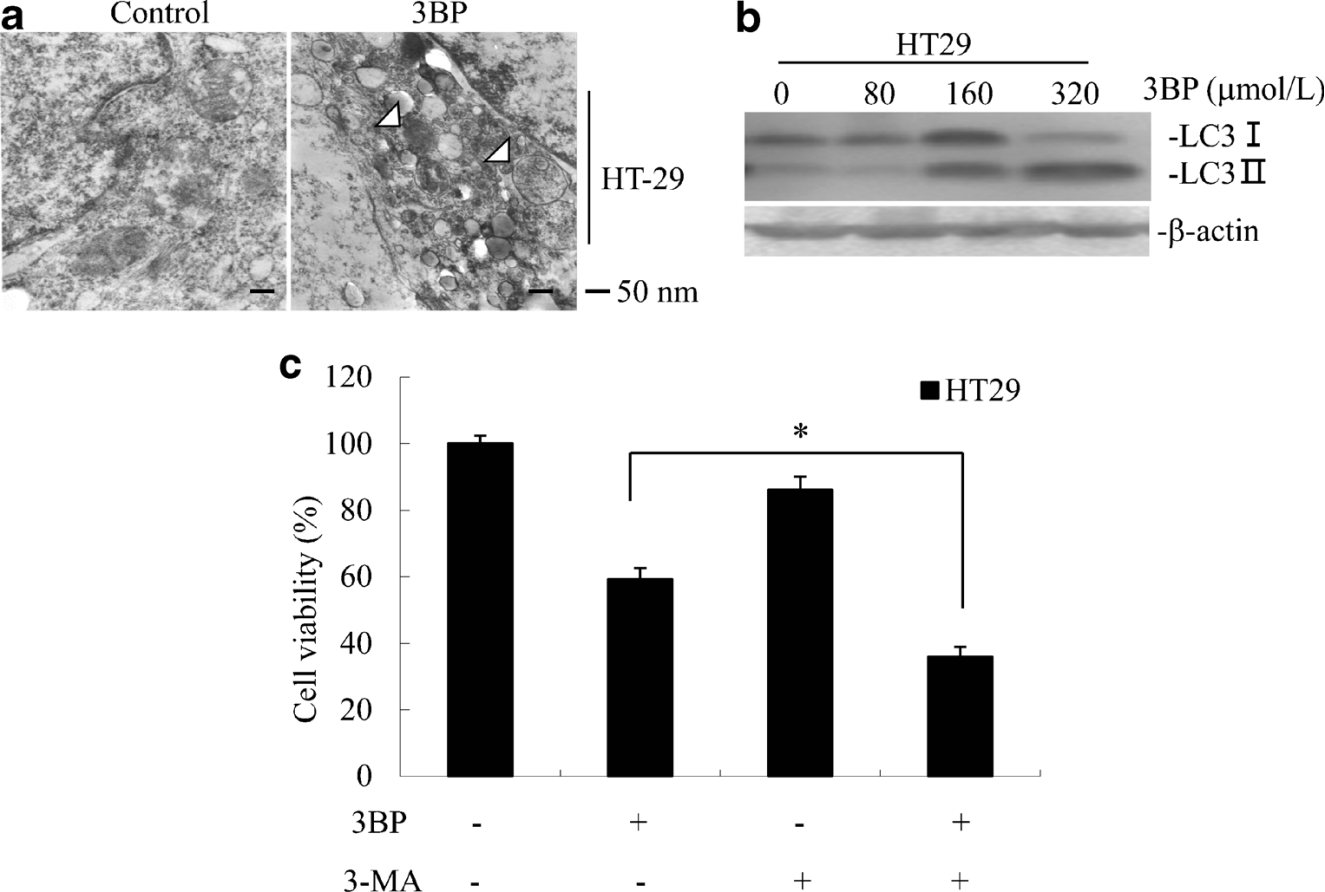
3BP-induced autophagy in colon cancer cells. **a** Electron micrographs of HT-29 cells treated for 24 h with 320 μmol/L 3BP or the vehicle dimethylsulfoxide (DMSO; control). *White arrowheads* indicate autophagosomes. **b** HT29 cells treated with 3BP (320 μmol/L) for 24 h were analyzed for the expression of autophagy-related proteins by western blotting, with β-actin serving as loading control. **c** HT29 cell viability following treatment with DMSO, 3BP (320 μmol/L), 3BP/3-MA (5 mmol/L), as analyzed by the MTT assay. Cells were pretreated with 3-MA 1 h before treatment with 3BP/3-MA. Data represent mean ± SEM of three independent experiments, *p < 0.05

Treatment with 3BP induced autophagy in HT29 cells, as evidenced by the appearance of autophagic vacuoles in the cytoplasm (Fig. 6a). The upregulation of LC3-II protein was also observed in 3BP-treated cells (Fig. 6b), indicating that autophagy was induced. Inhibiting autophagy with 3-MA enhanced cell death in 3BP-treated cells, suggesting that autophagy normally protects tumor cells against the cytotoxic effects of 3BP (Fig. 6c).

### 3BP treatment inhibits tumor growth in a mouse model

**Fig. 7:**
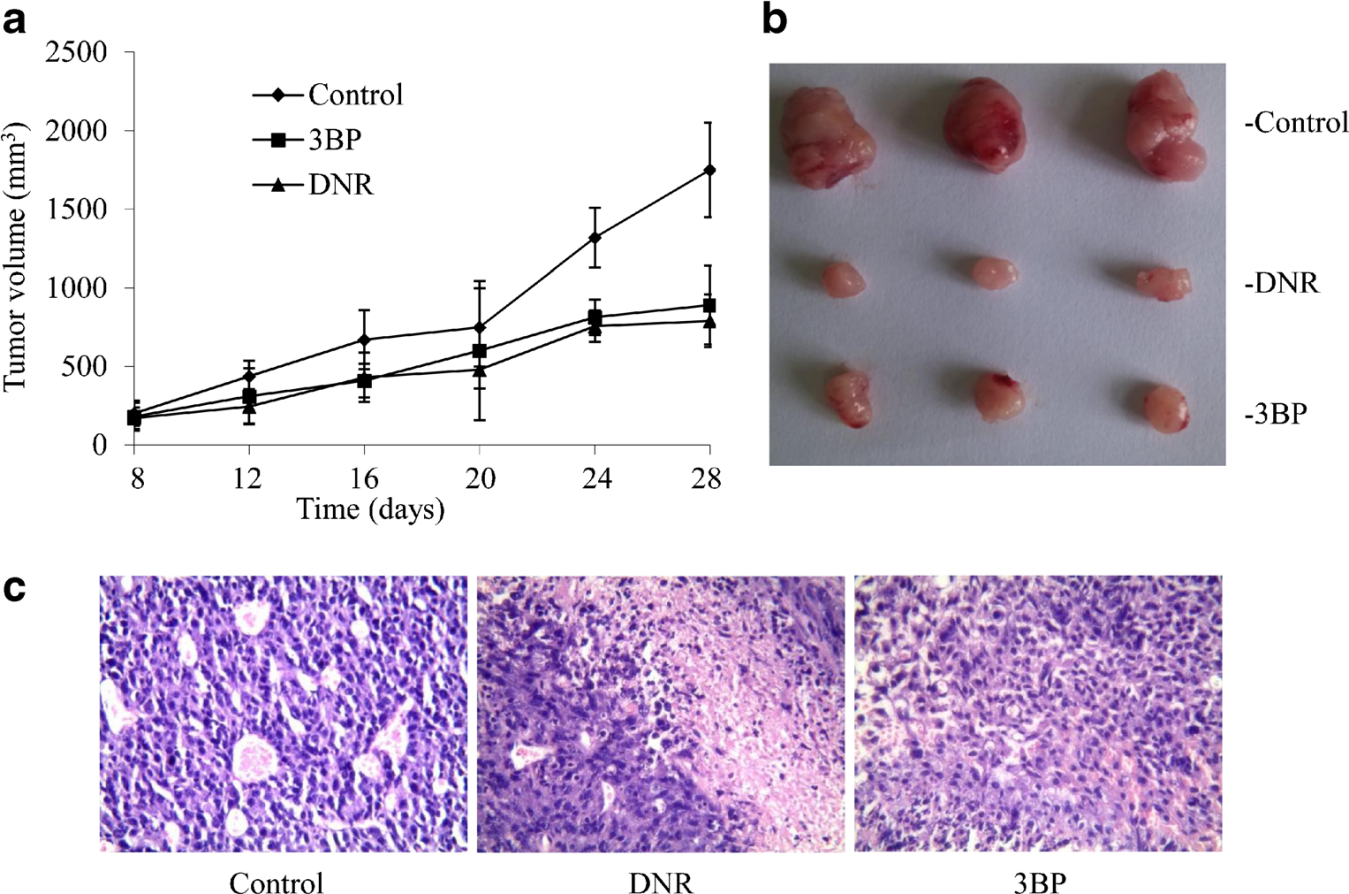
in vivo efficacy of 3BP in a nude mouse model. **a** SW480 cells were subcutaneously injected into the right flank of mice receiving 200 μL vehicle, 3BP (8 mg/kg), or DNR (0.8 mg/kg) treatment every 4 days by intraperitoneal injection. Mice were sacrificed after 28 days. **b** Representative tumors from each treatment group. Data represent mean ± SEM (*n* = 5). **c** H&E staining in vivo

The in vivo efficacy of 3BP was evaluated in a xenograft mouse model generated by injecting SW480 cells into nude mice. Treatment with PBS, 3BP (8 mg/kg) and daunorubicin (DNR; 0.8 mg/kg) was initiated when tumor volume reached approximately 100 mm^3^, 3BP prevented tumor growth (Fig. 7a). On day 28, the average tumor volumes in 3BP and DNR-treated groups (890 ± 260, and 790 ± 200 mm^3^, respectively) were significantly lower than in vehicle-treated control mice (1750 ± 374 mm^3^) (Fig. 7b). H & E staining of the tumors showed that there were rich blood vessels and large atypia nucleus in the tissue in control group, while chaotic distribution of blood vessel, karyopyknosis, inflammatory cell infiltration in experimental group, indicating 3BP induced extensive necrosis and inhibited tumor growth (Fig. 7c), providing evidence for its anti-tumorigenic action in vivo.

## Discussion

Tumorigenesis is characterized by mitochondrial dysfunction, hypoxia, tumor gene expression, and abnormal expression of metabolic enzymes (Warburg 1928). Tumor cells utilize ATP generated by glycolysis, which is less efficient than oxidative phosphorylation; as such, tumor cells consume more glucose (i.e., have higher glycolytic activity) than normal cells. As such, inhibiting glycolysis is a potential strategy for killing cancer cells (Simonnet et al. 2002).

As an inhibitor of glycolysis, 3BP is known to have an anti-tumorigenic function (Levy et al. 2012; Zhang et al. 2009b) in liver and breast cancers and other malignant tumors (Ganapathy-Kanniappan et al. 2010; Geschwind et al. 2002; Liu et al. 2009; Ota et al. 2013). We provide here the evidence for 3BP as an inhibitor colon cancer cell growth both in vitro and in vivo, which is accomplished by multiple cell death mechanisms, namely apoptosis, necroptosis, and autophagy (Clarke 1990). Programmed cell death is executed via specific intracellular biochemical pathways; apoptosis and necrosis are two such mechanisms that play significant roles in development and the maintenance of homeostasis in metazoans (Festjens et al. 2006), and their dysregulation is linked to various human diseases (Lockshin and Zakeri 2007). Necroptosis is a programmed and regulated form of necrotic cell death that is induced when a noxious stimulus is insufficient to trigger apoptosis in a cell. In such cases, tumor necrosis factor (TNF)-α activates TNF receptor (R) 1, which recruits RIP1 and other factors to form complex I, consisting of RIP1, RIP3, caspase-8, and Fas-associated death domain protein. These proteins dissociate from TNFR1 and RIP1 and are found in the cytosol as complex IIb, which is an effector of necroptosis (Lukens et al. 2013; Thapa et al. 2013). Caspase activation during apoptosis is accomplished through the extrinsic and intrinsic pathways. The former is activated by the binding of various cytokines such as TNF-α, TNF-related apoptosis-inducing ligand, and cluster of differentiation 95 to their respective receptors, leading to caspase-8 activation (Peter and Krammer 2003). The intrinsic pathway is activated following the release of proteins such as cytochrome c and Smac/Diablo from the mitochondrial intermembrane space into the cytosol, which then activate caspase-9 and relieve IAP-mediated inhibition of effector proteins (Du et al. 2000; Li et al. 1997; Verhagen et al. 2000). Activated caspase-8 and −9 subsequently cleave and activate several downstream caspases including caspase-3 and -7, which in turn cleave intracellular substrates, resulting in apoptosis.

Autophagy is a major intracellular pathway for the degradation and recycling of unused but long-lived proteins, damaged organelles, and invasive pathogens (Mizushima and Komatsu 2011) that functions in several organs, including the brain, heart, hematopoietic cells, and kidney (Komatsu et al. 2006; Mortensen et al. 2011; Takahashi et al. 2012). Mice lacking essential autophagy factors die within 1 day of birth (Kuma et al. 2004). Although it is essential for normal cell function and survival, autophagy can also be exploited by tumor cells for treatment resistance (Jin and White 2008; Mathew and White 2011). Activation of autophagy enhances cell survival (Narendra et al. 2008), and therefore combining standard chemotherapy with an autophagy inhibitor could potentially accelerate tumor cell death (Mathiasen and Jaattela 2002).

It is putative that proton-linked monocarboxylate transporters (MCTs) mediate 3BP uptake. The expression of MCT1 and MCT4 were decreased, possibly due to 3BP-induced cell death, cell membrane cracking, resulting in lower membrane transporters. We guess that inhibition of HKII by 3BP might decreases ATP levels, thereby blocking glycolysis in tumor cells and slowing or halting their growth.

The results of this study showed that 3BP has potent inhibitory effects on colon cancer tumor growth that are comparable to those of DNR. Interestingly, 3BP treatment induced autophagy in HT29 but not in SW480 cells; the reasons for this difference between cells lines is unclear, and will require further investigation. Moreover, the observation that the autophagy inhibitor 3-MA potentiated 3BP-induced cell death suggests that autophagy provides a survival advantage for energy-depleted tumor cells. For instance, the necroptosis inhibitor Nec-1 and z-VAD-fmk rescued cell viability that was reduced by 3BP treatment. If cellular energy shortage is exacerbated by suppressing the energy-generating glycolytic pathway in HT29 cells, it is possible that autophagy is insufficient to rescue cells from irreversible cell death but could enable tumor cells to survive.

The results of this study indicate that 3BP is a promising anti-tumorigenic drug that functions by activating multiple cell death pathways, and can potentially overcome tumor cell resistance to chemotherapeutic agents that activate a single cell death mechanism. Likewise, inhibiting glycolysis could be a point of anti-multi-resistant treatment, it remains to be subsequent research.

## Abbreviations

3BP: 3-Bromopyruvate
HK: Hexokinase
3-MA: 3-methyladenine
z-VAD-fmk: Caspase inhibitor benzyloxycarbonyl-Val-Ala-Asp fluoromethylketone
Nec-1: Necrostatin-1
CRC: Colorectal cancer
RIP1: Receptor-interacting protein 1
RIP3: Receptor-interacting protein 3
LC3: Microtubule-associated protein 1 light chain 3
MCTs: Monocarboxylate transporters
TNF: Tumor necrosis factor
MTT: 3-(4,5-dimethylthiazol-2-yl)-2,5-diphenyltetrazolium bromide
PI: Propidiumiodide
DAPI: 4′,6-diamidino-2-phenylindole
PBS: Phosphate Buffered Saline
DMSO: Dimethylsulfoxide
DMEM: Dulbecco’s Modified Eagle’s Medium
DNR: Daunorubicin
